# The Temperature-Regulation of *Pseudomonas aeruginosa cmaX-cfrX-cmpX* Operon Reveals an Intriguing Molecular Network Involving the Sigma Factors AlgU and SigX

**DOI:** 10.3389/fmicb.2020.579495

**Published:** 2020-10-21

**Authors:** Emeline Bouffartigues, Ishac Si Hadj Mohand, Olivier Maillot, Damien Tortuel, Jordane Omnes, Audrey David, Ali Tahrioui, Rachel Duchesne, Cecil Onyedikachi Azuama, Michael Nusser, Gerald Brenner-Weiss, Alexis Bazire, Nathalie Connil, Nicole Orange, Marc G. J. Feuilloley, Olivier Lesouhaitier, Alain Dufour, Pierre Cornelis, Sylvie Chevalier

**Affiliations:** ^1^Laboratoire de Microbiologie Signaux et Microenvironnement (LMSM) EA 4312, Normandie Université, Université de Rouen Normandie, Centre de Sécurité Sanitaire de Normandie, Evreux, France; ^2^Institute of Functional Interfaces, Karlsruhe Institute of Technology, Karlsruhe, Germany; ^3^Laboratoire de Biotechnologie et Chimie Marines (LBCM) EA3884, IUEM, Université de Bretagne-Sud, Lorient, France

**Keywords:** *Pseudomonas aeruginosa*, temperature, cell wall stress ECF sigma factor, regulation of transcription, membrane fluidity

## Abstract

*Pseudomonas aeruginosa* is a highly adaptable Gram-negative opportunistic pathogen, notably due to its large number of transcription regulators. The extracytoplasmic sigma factor (ECFσ) AlgU, responsible for alginate biosynthesis, is also involved in responses to cell wall stress and heat shock via the RpoH alternative σ factor. The SigX ECFσ emerged as a major regulator involved in the envelope stress response via membrane remodeling, virulence and biofilm formation. However, their functional interactions to coordinate the envelope homeostasis in response to environmental variations remain to be determined. The regulation of the putative *cmaX-cfrX-cmpX* operon located directly upstream *sigX* was investigated by applying sudden temperature shifts from 37°C. We identified a SigX- and an AlgU- dependent promoter region upstream of *cfrX* and *cmaX*, respectively. We show that *cmaX* expression is increased upon heat shock through an AlgU-dependent but RpoH independent mechanism. In addition, the ECFσ SigX is activated in response to valinomycin, an agent altering the membrane structure, and up-regulates *cfrX-cmpX* transcription in response to cold shock. Altogether, these data provide new insights into the regulation exerted by SigX and networks that are involved in maintaining envelope homeostasis.

## Importance

Because temperature is one of the first physical signal that allows bacteria to distinguish a potential host from its environment, the response of *P. aeruginosa* has been more investigated in case of elevated rather than low temperature. Here, we show that the ECFσ SigX is an effector in the latter condition, which controls the transcription of the two members of an intriguing operon encoding the mechanosensitive channel CmpX and the hypothetical protein CfrX, while *cmaX* is under the control of AlgU. In addition, we show that SigX is activated following membrane structure alterations. More generally, we have identified temperature as a key environmental factor regulating antagonistically the expression and the activity of the two major cell wall stress response ECFσ in *P. aeruginosa*, suggesting that they are involved in specific but interconnected networks, in which the AlgU-dependent global transcriptional regulator AmrZ (alginate and motility regulator Z) could play a role by a mechanism that remains to be explored.

## Introduction

Temperature shift is a ubiquitous signal perceived by living organisms to adapt their behaviors. The heat shock (HS) and cold shock (CS) responses (R), involve heat shock proteins (Hsps), and cold inducible proteins (Cips), respectively. Importantly, HS and CS pathways are also activated in response to antibiotics in bacteria (VanBogelen and Neidhardt, 1990; Kindrachuk et al., 2011), highlighting their importance in antibiotic resistance, noticeably of ESKAPE pathogens (*Enterococcus faecium, Staphylococcus aureus, Klebsiella pneumoniae, Acinetobacter baumanii, Pseudomonas aeruginosa*, and *Enterobacter* species) (Esposito and De Simone, 2017). *P. aeruginosa* is a highly adaptable Gram-negative bacterium that can colonize a variety of ecological niches (Spiers et al., 2000) and infect various hosts (Rahme et al., 2000; Baldini et al., 2014). Being intrinsically resistant to a wide range of antibiotics and disinfectants, *P. aeruginosa* is responsible of both acute and chronic community- and hospital-acquired infections (Lyczak et al., 2002). Its remarkable versatility is associated with the over-representation of genes encoding sensors, signal transduction systems and regulators such as sigma factors (Stover et al., 2000; Chevalier et al., 2018).

A sudden temperature rise above 45°C activates the RpoH (σ^32^)-dependent HS response in *P. aeruginosa* (Allan et al., 1988) that, together with the extracytoplasmic sigma factor (ECFσ) AlgU, co-regulates the production of the alginate exopolysaccharide via over-expression of the *algD* operon leading to a mucoid phenotype (Schurr and Deretic, 1997). AlgU is a homolog of the *Escherichia coli* RpoE cell wall stress response ECFσ, whose expression is auto-regulated at the transcriptional level, and its activity modulated at the post-translational level by a conserved mechanism of regulated-intramembrane proteolysis (Chevalier et al., 2018). The AlgU regulon includes *rpoH* encoding the heat shock RpoH sigma factor (Schurr et al., 1995), *amrZ* (AlgU-dependent alginate and motility regulator Z) encoding a transcriptional regulator that activates the expression of genes involved in motility and in alginate production, including in particular *algD* (Tart et al., 2006), *oprF* encoding the major and multi-functional outer membrane porin (Chevalier et al., 2017), together with numerous genes involved in bacterial adaptation and cell wall synthesis (Wood and Ohman, 2009; Schulz et al., 2015). In agreement with its role in envelope homeostasis, AlgU is more expressed in response to hyperosmolarity (Aspedon et al., 2006), inhibition of cell wall synthesis (Wood and Ohman, 2009), exposure to microgravity stress (Crabbe et al., 2010), the absence of the Hfq RNA chaperone (Sonnleitner et al., 2006) and OprF (Bouffartigues et al., 2015). Except for hyperosmolarity, the same conditions also increase the expression of *sigX*, another envelope stress ECFσ, which responds to stimuli via unknown regulatory mechanisms. SigX is up-regulated in LB containing high sucrose concentration (Bouffartigues et al., 2014) and by hypo-osmolarity (Brinkman et al., 1999; Bouffartigues et al., 2012), two conditions affecting membrane homeostasis. Initially, SigX was shown to be involved in *oprF* transcription together with AlgU. However, the characterization of the transcriptome and proteome of *sigX* mutant and *sigX* overexpressing strains suggested its involvement in the expression of fatty acids biosynthetic genes (Gicquel et al., 2013; Blanka et al., 2014; Fléchard et al., 2018). Although a SigX-overexpressing strain is more resistant to a 2 h CS at 15°C (Boechat et al., 2013), highlighting its role in membrane fluidity, its expression in response to temperature variations has not been investigated.

In addition to OprF (Jaouen et al., 2004), the products of at least two genes located immediately upstream *sigX*, *cmaX* and *cmpX*, could be involved in temperature adaptation. Indeed, CmaX is homologous to the CorA Mg^2+^-dependent channel. Interestingly, the increase of these transport proteins enhanced the *Salmonella enterica* survival at high temperature (O’Connor et al., 2009), a condition that increased transcription of both *Escherichia coli corA* (Richmond et al., 1999) and *P. aeruginosa cmaX* (Chan et al., 2016). CmpX is predicted to be a small mechanosensitive ion channel (MscS). These membrane tensions-gated pores are involved in osmotic downshift adaptation (Martinac et al., 2014). Inversely to *corA*, the expression of *mscS* in *E. coli*, increased in response to low temperature (White-Ziegler et al., 2008). In addition, the MscS mechanosensitive properties are affected by temperature (Koprowski et al., 2015), suggesting that these proteins could be membrane fluidity sensors. *cmaX* and *cmpX* are predicted to form an operon together with *cfrX* (Database for prokaryotic OpeRons DOOR) (Mao et al., 2009). The latter gene is predicted to encode a small hypothetical protein of 84 amino acids, the cellular location and function of which have not yet been demonstrated, however, *cfrX*, like *cmpX*, have been shown to belong to the SigX regulon (Gicquel et al., 2013; Schulz et al., 2015). Altogether, these observations suggest that this putative operon might be differentially regulated by temperature.

Here, we studied the impact of temperature shifts on the *cmaX-cfrX-cmpX* operon expression and investigated the involvement of the sigma factors AlgU and SigX in its regulation. We showed that this operon expression is induced under HS from a *cmaX*-proximal promoter region regulated by AlgU and under CS from a SigX-dependent promoter upstream *cfrX*. In connection with this observation we also demonstrated that the level of *sigX* mRNA was increased following an exposure to valinomycin, a membrane modification promoting agent, suggesting that SigX responds to membrane alteration. Interestingly, we observed that SigX is involved in the regulation of *amrZ* in response to CS. Finally, our data suggest a fine-tuned interconnection between the two cell wall stress response SigX and AlgU ECFσ that could be relevant in others stress conditions affecting the membrane fluidity and structure.

## Results

### Members of the *cmaX-cfrX-cmpX* Operon Are Differentially Regulated in Response to HS or CS

**FIGURE 1:**
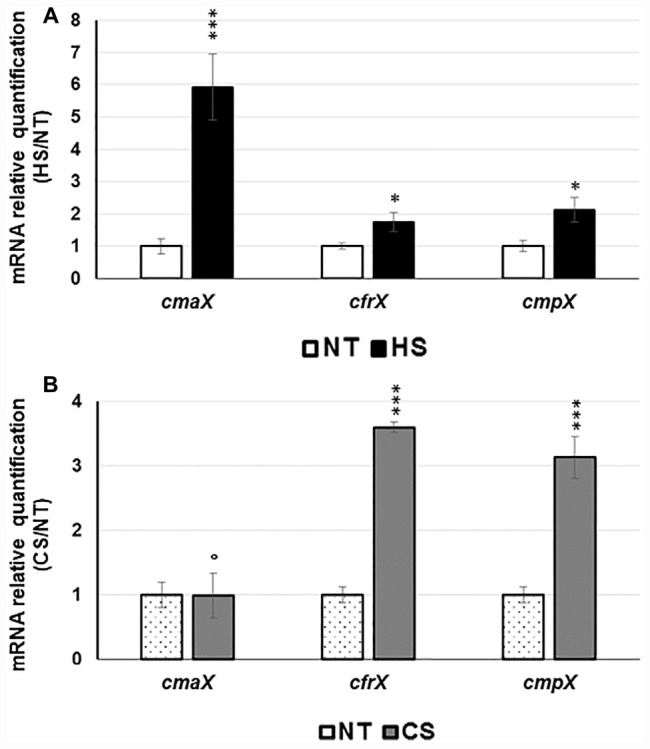
Effect of HS and CS on *cmaX-cfrX-cmpX* expression *P. aeruginosa* H103. Histograms show the means of normalized expression for each gene in HS **(A)** or CS **(B)** relatively to the control condition at 37°C as calibrator. Error bars indicate SEM of mean, *n* = 4. Significant differences between the sets of normalized expression obtained in each stress condition were calculated against those obtained at 37°C using a two-tailed *t*-test, and *P-*value are indicated by * or *** if < 0.05 or 0.0001, respectively. The symbol ° is indicated for non-significant results.

To study the effects of temperature on the expression of the *cmaX-cfrX-cmpX* operon, we chose to expose the strain H103 to thermal down- and up-shifts from 37°C without causing lethal cell damages. To this aim, H103 cells were grown at 37°C until the mid-log phase, before being exposed to sudden shifts at 4°C or at 60°C, during 15 or 30 min, respectively. Then, the bacterial cell viability was evaluated by flow cytometry after a double labeling with SYTO 9 (alive cells) and propidium iodide (dead cells) (Supplementary Figure S1). Compared to the control condition at 37°C, a downshift at 4°C during 15 or 30 min had no significant effect on H103 wild-type [(WT); Hancock and Carey, 1979] viability while an upshift at 60°C increased the percentage of dead cells from 0.6 to 1.9% after 15 min and from 0.3 to 2.4% after 30 min of exposure. Noticeably, the bacterial population submitted to a 30 min HS was more heterogeneous compared to 15 min exposure, with 18.1 and 11.8% of injured cells, respectively. Therefore, the following sublethal conditions (HS: 15 min at 60°C; CS: 30 min at 4°C) were applied in the subsequent experiments. The impact of these temperature shifts on the expression of the predicted *cmaX-cfrX-cmpX* operon was evaluated using RT-qPCR. Following HS, *cmaX*, *cfrX*, and *cmpX* mRNA levels were increased (Figure 1A and Supplementary Table S1) with *cmaX* transcripts being the most elevated. Conversely, only *cfrX* and *cmpX* were significantly up-regulated under CS (Figure 1B and Supplementary Figure S1). These results suggest that *cfrX-cmpX* could be thermo-regulated at the transcriptional level from a promoter lying upstream of *cfrX*.

### Identification of a Promoter Region Upstream of *cfrX-cmpX*

**FIGURE 2:**
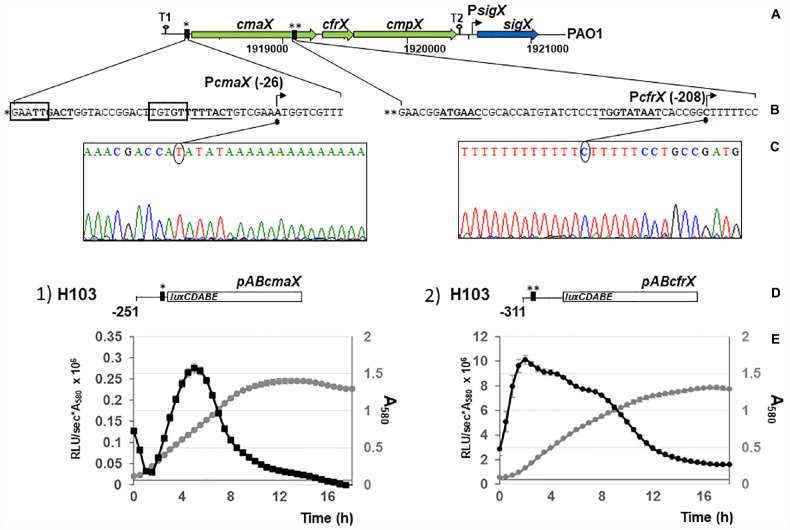
At least two promoter regions control the transcription of *cmaX, cfrX*, and *cmpX* in *P. aeruginosa* H103. **(A)** Schematic representation of the genomic environment of the predicted operon *cmaX-cfrX-cmpX* of *P. aeruginosa*. Large arrows indicate the direction of transcription of the ORFs, loops show the position of the putative rho-independent transcription terminators (T1 and T2), and the vertical black rectangles represent the two BPROM predicted promoters. **(B)** Sequences of the two putative promoters P*cmaX* and P*cfrX*. Predicted –35 and –10 motifs using BPROM are in bold case and underlined, the DNA-binding motif for AlgU is surrounded in black and TSSs are indicated by arrows. **(C)** Electrophoregrams obtained from 5′RACE-PCR experiments confirming the promoters upstream *cmaX* and *cfrX* genes. **(D)** Schematic representation of the construct harboring the pAB*cmaX* or pAB*cfrX* vectors. **(A–C)** * and ** correspond to the two distinct promoter regions. **(E)** Relative activity of the promoter regions upstream *cmaX* or *cfrX* in *P. aeruginosa* H103 during growth in LB medium at 37°C. Reporter constructs contain the P*cmaX* (construct 1, pAB*cmaX*) or the P*cfrX* (construct 2, pAB*cfrX*) promoter fused to the *lux*CDABE operon. The large white rectangle indicates the *lux*CDABE cassette, the vertical black rectangles represent promoters and a line shows the DNA sequence directly upstream *cmaX* or *cfrX* ORF. The position of the last nucleotide (relatively to the translational initiation site) included within the cloned DNA fragment is highlighted. The mean profile for growth and reporter assay from H103 transformed with construct 1 or 2 are shown and correspond to 3 independent experiments, ± SD. Transformants growth is presented in gray and the transcriptional activity pattern in black.

The 500 nucleotides DNA sequences upstream of each gene were analyzed using Bprom (Softberry). Two s70-like consensus promoters were predicted (Figures 2A,B), TTGACT(-35)-N17-TTTACT(-10) located in the intergenic region between *rraA* (PA1772) and *cmaX*, and ATGAAC(-35)-N17-TGGTATAAT(extended-10), within the *cmaX* encoding sequence, upstream of *cfrX*. No s70-like promoter was predicted within the 500 nucleotides upstream of *cmpX*. The consensus sequence of SigX-dependent promoters remains not precisely defined (Schulz et al., 2015) and we therefore could not search putative promoters by bioinformatic analysis. Using a 5′ RACE-PCR assay, the transcriptional start sites (TSS) of *cmaX* and *cfrX* were then identified at positions −26 for *cmaX* and −208 for *cfrX* relatively to the translational initiation site, downstream of the corresponding predictive −35 and −10 boxes (Figures 2B,C). The activity of each promoter region was measured during *P. aeruginosa* H103 growth in LB medium at 37°C using two bioluminescent reporters, pAB*cmaX* and pAB*cfrX* (Figures 2D,E). The relative luminescence (RL) signal from pAB*cmaX* decreased at the beginning of the mid-log phase before increasing and finally reaching its maximum level of 0.27 RLU/sec^∗^A_580_ × 10^6^ before decreasing at the end of the log phase. The pAB*cfrX* promoter region was much more active, reaching a maximal level of 9.8 RLU/sec^∗^A_580_ × 10^6^. The RL increased rapidly at the beginning, decreased slowly during the log phase and more steeply during the stationary phase. Confirming our sequence analysis, no transcriptional activity was detected from a transcriptional fusion harboring a 224 bp DNA fragment upstream of *cmpX* (data not shown). Our data confirmed the existence of a promoter region, which could differentially regulate the expression of *cfrX* and *cmpX* compared to that of the full-length *cmaX-cfrX-cmpX* operon in different environmental conditions.

### AlgU Is Involved in the Regulation of *cmaX-cfrX-cmpX*

**FIGURE 3:**
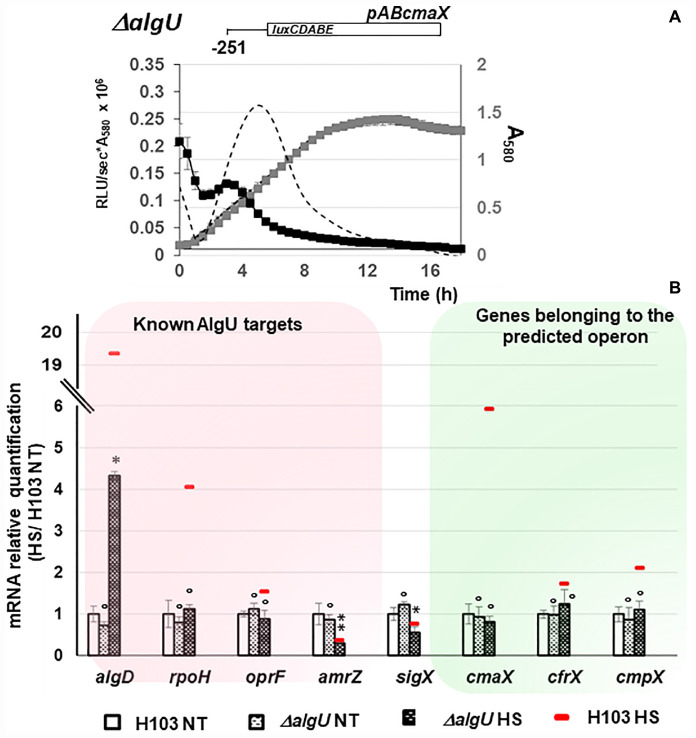
Involvement of AlgU in *cmaX, cfrX, and cmpX* regulation under HS. **(A)** Activity pattern of the promoter region upstream *cmaX* in an *algU* mutant (black squares) or in H103 (dashed line) during growth in LB at 37°C of the H103 wild-type strain (black dashed line) or Δ*algU* mutant (gray squares). *n* = 3, error bars indicate SD. **(B)** Histograms show the means of normalized expression relatively to H103 at 37°C as calibrator (white bars) for some known *algU* targets (red frame) and the studied operon (green frame) in H103 (red line) and in Δ*algU* mutant exposed (dashed back bars) or not (dashed white bars) to HS. NT, not treated. Errors bars indicate SEM, *n* = 4. Significant differences between the sets of normalized expression obtained in HS condition were calculated against those obtained at 37°C for H103 using a two-tailed *t*-test and *P-*value, which are indicated by *, **, if < 0.05, 0.001, respectively. The symbol ° is indicated for non-significant results.

**FIGURE 4:**
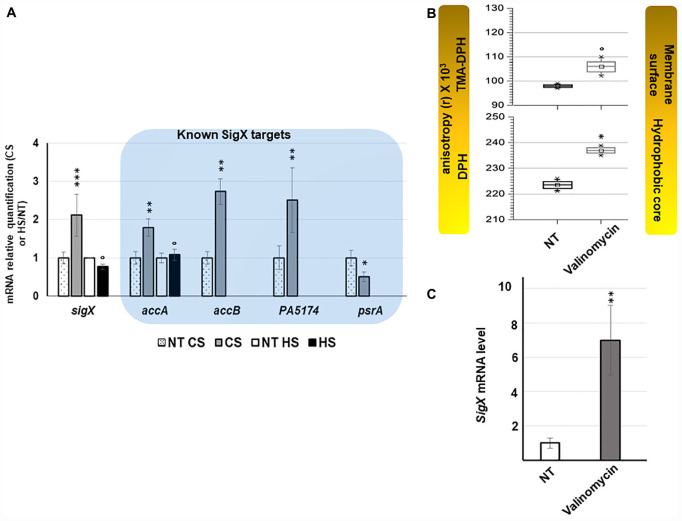
*sigX* is up-regulated in response to CS and valinomycin promoting membrane stiffening. **(A)** Effect of thermal shocks on *sigX* expression and activity. Gray and black bars represent the mean normalized expression for *sigX* and its targets after CS or HS relatively to those obtained for H103 at 37°C (dotted and white bars). NT, not treated. Errors bars indicate SEM, *n* = 4. Significant differences between the sets of normalized expression obtained in each stress condition were calculated against those obtained at 37°C using a two-tailed *t*-test and *P*-value, which are indicated by *, **, or *** if < 0.05, 0.001, or 0.0001, respectively. The symbol ° is indicated for non-significant results. **(B)** TMA-DPH and DPH anisotropies was used to evaluate the effect of 45 μM valinomycin on membrane fluidity 2 h after the addition of the membrane acting-agent to mid-log phase cultures. Values obtained from unexposed cultures are indicated NT: not treated. Box plots correspond to each sets of data from three independent experiments. Means, medians and min/max anisotropies values are indicated by squares, lines and crosses, respectively. Significant differences between each condition were calculated using a two-tailed *t*-test and *P*-value, which are indicated by * if < 0.05. The symbol ° is indicated for non-significant results. **(C)** Relative expression of *sigX* after 2 h of exposure to 45 μM valinomycin. NT, not treated. Error bars indicate SEM, *n* = 3, *P*-value is calculated by two-tailed *t*-test and is indicated by ** if < 0.001.

The expression of *algU* and its known targets *algD*, *rpoH*, *oprF*, and *amrZ* was quantified using RT-qPCR. The *algU* mRNA level was increased when bacteria were under HS (Supplementary Figure S2 and Supplementary Table S1). Accordingly, the mRNA levels of *algD*, *rpoH*, and *oprF* were significantly increased, while the expression of *amrZ* was reduced. The expression of the same genes was not significantly changed in response to a CS (Supplementary Figure S2 and Supplementary Table S1), confirming the involvement of *P. aeruginosa* H103 AlgU in the HS, but not in the CS response. By visual inspection, a putative AlgU recognition motif was detected [GAATT(-35)-N15-TGTGT(-10)] upstream of *cmaX* (Figure 2B), which is highly similar to the known AlgU consensus binding sequence (GAACTT-N16/17-TCTNN) (Firoved et al., 2002). To confirm the involvement of AlgU in this promoter region activity, the pAB*cmaX* plasmid was introduced in an isogenic *algU* mutant and the bioluminescence signal was measured during growth in LB medium at 37°C (Figure 3A). The RL signal reached a maximum level of 0.21 RLU/sec^∗^A_580_ × 10^6^ at the beginning of the mid-log phase, corresponding to an increase of about 50% compared to the WT, followed by a 50% decrease during exponential growth, suggesting an AlgU involvement in the control of this promoter region at this growth phase. The involvement of AlgU in the HS-inducible expression of the previously selected AlgU-regulated genes and of the *cmaX-cfrX-cmpX* operon was examined using RT-qPCR. Relatively to the expression measured in a WT strain similar expression levels were observed in the *algU* mutant grown at 37°C for all the genes tested, suggesting that AlgU only slightly contributes to their transcription in our control condition (Figure 3B and Supplementary Table S1). Although a residual heat-inducible expression of *algD* of about 30% was observed in the *algU* mutant, the *rpoH* and *oprF* expressions were unchanged when the *algU* mutant strain was exposed to HS, confirming that AlgU was required for their increased transcription in response to HS (Figures 3B, 6 and Supplementary Table S1). Interestingly, as observed for H103, the *amrZ* expression was down-regulated in the HS exposed *algU* mutant, suggesting an AlgU-independent mechanism of *amrZ* repression under HS. Similar to AlgU-targets *rpoH* and *oprF*, the heat-inducible expression of *cmaX-cfrX-cmpX* measured in H103 under HS was not observed in an *algU* mutant, indicating an AlgU involvement in the HS-induced increased expression of the operon (Figure 3B and Supplementary Table S1). To evaluate the involvement of RpoH in the heat-inducible expression of the operon a *rpoH* mutant in PAO1 was used. As previously observed in H103, the expression of *cma*X was increased and in a lesser extent, those of c*frX* and *cmpX* in PAO1 exposed to our HS condition (Supplementary Figure S3 and Table 1). A similar effect was observed in a *rpoH* mutant, suggesting that RpoH is not involved in the regulation of the operon in PAO1. Proteomic analysis of H103 under CS Total proteins from H103 cells harvested during mid-log phase and exposed or not to CS during 30 min were extracted and separated by two-dimensional gel electrophoresis (2D-E). Nine protein spots showing reproducible different intensities were identified by mass spectroscopy, among which some correspond to well-known markers of the CS response in other bacterial species (Beran and Simons, 2001; Gao et al., 2006; Table 1, Supplementary Figure S4, and Supplementary Table S2): two translation elongation factors (EF-Tu and Ef-Ts) and a well-described cold-inducible RNase (PNP)were up-regulated, and could together contribute to facilitate gene expression at the post-transcriptional level. Five of the identified proteins are involved in general metabolism and energy production. The arginine deiminase (ArcA), of the arginine fermentation pathway was down-regulated, while the 3-phosphoglycerate kinase (Pgk), the 4-hydroxyphenyl-pyruvate dioxygenase (Hpd), the electron transfer flavoproteins EtfA and EtfD were more abundant under CS, respectively (Table 1). Pgk, Hpd, and the Etf pathway contribute to oxidative energy production through their involvement in glycolysis, the phenylalanine/tyrosine catabolism and the utilization of branched-chain amino acids and fatty acids, respectively. In agreement with membrane lipid composition remodeling in response to CS, the abundance of the acetyl-coenzyme carboxylase A (AccA) was higher under CS (Figure 4 and Table 1). This protein is a subunit of the ACC complex and acts in concert with AccB, AccC, and AccD to provide the metabolic intermediate malonyl-CoA for *de novo* biosynthesis of fatty acids, which could contribute to restore the membrane fluidity upon a temperature downshift. Interestingly, the long chain fatty acid sensory regulator PsrA which negatively regulates the genes encoding the Etf pathway (Kojic et al., 2005; Kazakov et al., 2009) and *acc* genes, were previously shown to belong to the SigX regulon (Boechat et al., 2013; Gicquel et al., 2013; Blanka et al., 2014), suggesting that this ECFσ is able to respond to a sudden temperature downshift. The mRNA levels of *accA* and *accB* under CS condition were compared to those at 37°C in the WT strain (Figure 4A). In line with the increased abundance of AccA, *accA* and *accB* expression was increased. Although a decrease of the *accB* expression was previously shown to increase the c-di-GMP intracellular level *via* the Wsp system (Blanka et al., 2014), our CS conditions did not affect the c-di-GMP intracellular level (Supplementary Figure S5). Interestingly, the self-regulated *psrA* gene was down-regulated, indicating possibly a transcriptional de-repression of the Etf pathway (Figure 4). The observed positive effect of the temperature downshift on *accA* and *accB* mRNA abundances could result from the accumulation of SigX since its expression was up-regulated as well (Figure 4A). Consistently, CS caused an increase of the mRNA level of *fabY* (PA5174), a presumed direct SigX-target encoding the predominant FabY enzyme catalyzing the condensation of acetyl coenzyme A with malonyl-ACP (Yuan et al., 2012).

**TABLE 1:** List of identified proteins differentially expressed under CS in *P. aeruginosa* H103.

Pseudocap	Spot number	PA number	Protein name	Putative function	Fold change CS/Control	Standard deviation
Energy metabolism	1	PA2951	EtfA	Electron transfer flavoprotein, alpha subunit	2.33	0.3
	2	PA0552	Pgk	3-phosphoglycerate kinase	3.93	0.16
	3	PA2953	EtfD	Electron transfer flavoprotein-ubiquinone oxidoreductase	2.85	0.5
Amino acid biosynthesis and metabolism	4	PA5171	ArcA	Arginine deiminase	0.29	0.14
	5	PA0865	Hpd	4-hydroxyphenylpyruvate dioxygenase	3.25	1.43
Fatty acid and phospholipid metabolism	6	PA3639	AccA	Acetyl-coenzyme A carboxylase carboxyl transferase (alpha subunit)	3.21	0.44
Translation, post-translational modification, degradation	7	PA3655	Tsf	Elongation factor Ts	1.69	0.03
	8	PA4265	TufA	Elongation factor Tu	2.43	0.18
	9	PA4740	Pnp	Polyribonucleotide nucleotidyltransferase	1.61	0.09

*Spots with an average fold change above 1.5 were considered. Standard deviations were calculated from three biological replicates.*

### Valinomycin Increases *sigX* Expression

To evaluate whether induced stiffening of the membrane could represent a stimulus increasing *sigX* expression, its mRNA level was measured after treatments of cells with sublethal concentrations of valinomycin, an antibacterial agent affecting the membrane. Alterations of membrane fluidity were evaluated by fluorescence anisotropy at the aqueous interface (1-[4-(trimethylamino)phenyl]-6-phenyl-1,3,5-hexatriene (TMA-DPH)) and at the hydrophobic core (1,6-diphenyl-1,3,5-hexatriene (DPH)) of the membrane. As previously reported (El Khoury et al., 2017) a significant increase of the anisotropy of both TMA-DPH and DPH probes was observed for valinomycin-exposed cells, confirming that valinomycin promotes membrane stiffening (Figure 4B and Supplementary Figure S6). Shorter exposition times were used to follow the early response of *sigX* to membrane perturbation (Figure 4C). After 2 h promoting the incorporation of valinomycin into the lipid bilayer, an increase of fluorescence anisotropy was observed with both membrane probes, showing that the membranes were more ordered and compact in this culture condition (Figure 4B) while a strong increase of *sigX* mRNA level was measured (Figure 4C). Taken together, this set of data shows that *sigX* responds to membrane stressors causing membrane stiffening (Figure 6).

### SigX Is Involved in the Expression of *cfrX-cmpX* and *amrZ* Under CS

**FIGURE 5:**
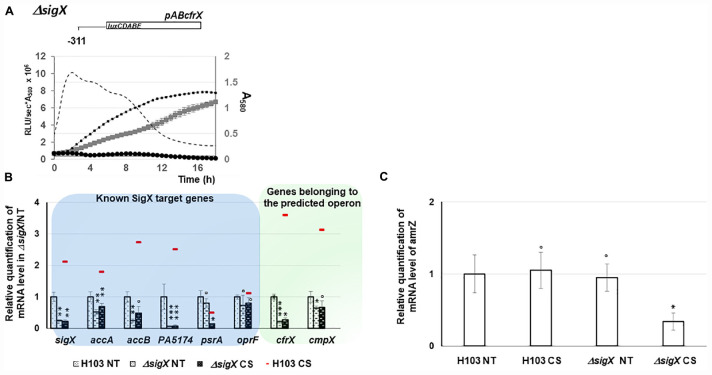
Involvement of SigX in *cmaX*, *cfrX*, and *cmpX* and *amrZ* regulation under CS. **(A)** Growth of H103 (black squares, dashed line) and PAOSX *sigX* mutant (gray squares) in LB at 37°C and activity pattern of the promoter region upstream *cfrX* in H103 (dashed line) and PAOSX (black circles) background. *N* = 3, error bars indicate SD. **(B)** Means of normalized expression relatively to H103 at 37°C (dotted white bars) of *sigX* and its targets (blue frame) and genes belonging to the operon (green frame), in H103 (red line) and in PAOSX exposed (dashed back bars) or not (dashed white bars) to CS. NT, not treated. **(C)** White bars show mean normalized *amrZ* expression (relative to H103 at 37°C) in H103 or PAOSX exposed or not to CS. **(B,C)** Error bars indicate SEM for *n* = 4, *P*-value were calculated by two-tailed *t*-test and are indicated by *, **, or *** if < 0.05, 0.001, or 0.0001, respectively. The symbol ° is indicated for non-significant results.

When comparing the relative bioluminescence signals measured from H103 or Δ*sigX* (PAOSX) harboring pAB*cfrX*, only a residual background activity of about 8% was observed for Δ*sigX* during the log phase, revealing a major contribution of SigX to *cfrX* promoter activity (Figure 5A). The expression of the set of previously identified SigX targets was further evaluated in PAOSX at 37°C and under CS by RT-qPCR. Except for *psrA*, the expression of *sigX* as well as of the other SigX-regulated genes, *accA*, *accB* and PA5174 (*fabY*), was significantly down-regulated in Δ*sigX* compared to H103 (Figure 5B and Supplementary Table S1). Confirming that *cfrX* and *cmpX* are under the control of SigX in our control condition at 37°C, their expression was likewise down-regulated. Under CS, the mRNA levels of these genes, including *cfrX* and *cmpX*, were not significantly increased in Δ*sigX* (Figure 5B and Supplementary Table S1), confirming that their cold-inducible expression was SigX-dependent. The *psrA* mRNA level was lower in Δ*sigX* than in H103 when cells were exposed to a sudden temperature downshift (Figure 5B and Supplementary Table S1). This result might indicate that the repression of *psrA* in response to CS is likely related to a SigX-independent mechanism. Taken together, these data support that the *cfrX-cmpX* transcriptional unit is under the direct control of SigX, which is responsible for the increase of its transcription in response to CS.

**FIGURE 6:**
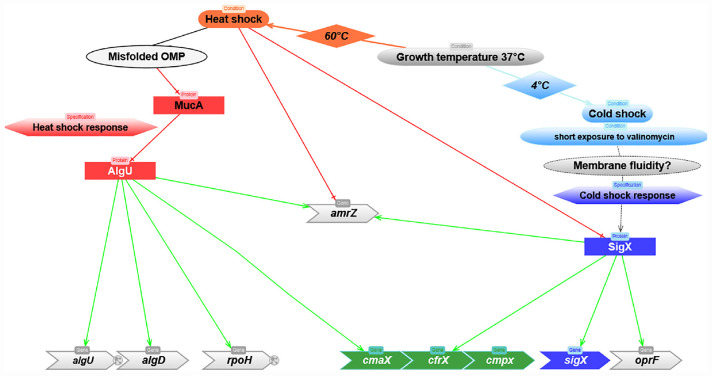
Predicted model depicting the molecular mechanisms involved in the regulation of *cmaX-cfrX-cmpX* in response to HS and CS. Red and green lines correspond to negative or positive regulation, respectively.

We next focused on AmrZ, whose expression was co-regulated with *sigX* during HS (Figure 3B), and which was shown by RNA-seq analysis to positively control *cfrX* and *cmpX*, as well as the CS-inducible SigX-target genes *accA* and PA5174 (*fabY*) (Jones et al., 2014). Consistently, AmrZ binding was identified upstream of *sigX* by ChIP-Seq in *P. fluorescens* F113 genome (Martínez-Granero et al., 2014). Altogether this set of data suggests that SigX and AmrZ could be involved in a feedback loop depending on the environmental condition. To evaluate the contribution of SigX in *amrZ* transcription in response to CS, the *amrZ* mRNA level was measured in the WT and Δ*sigX* strains exposed or not to CS. In H103, the *amrZ* expression was not upregulated in response to CS (Figure 5C). However, it was about three times down-regulated in PAOSX exposed to CS compared to untreated and CS treated H103, indicating that SigX could be involved in *amrZ* transcription in response to CS (Figure 6).

## Discussion

Sudden temperature variations can trigger a cell wall stress response (CWSR) by altering the membrane composition, structure and functionality. Although the molecular mechanisms involved in the HS response are relatively well characterized in *P. aeruginosa*, involving the AlgU ECFσ via its regulon member RpoH, those involved in the CS response have only been scarcely described. Therefore, characterizing these mechanisms should highlight new effectors playing a key role in envelope homeostasis and in the adaptive capacity of *P. aeruginosa* under other chemical and physical stresses. In the present study, we focused on the description of the uncharacterized *cmaX-cfrX-cmpX* operon regulatory mechanisms by a combination of approaches, including bioinformatics, transcriptomics, proteomics and fluorescence polarization assays. Based on the identification of two promoter regions located upstream of *cmaX* and *cfrX-cmpX*, we demonstrated that AlgU and SigX differentially regulate the members of this cluster in function of temperature variations, and we therefore postulate that these two CWSR ECFσ respond to specific stimuli that can be caused by opposite temperature variations from 37°C (Figure 6). In agreement with previous results obtained by genome-wide transcription start site (TSS) mapped by RNA-seq (Wurtzel et al., 2012; Schulz et al., 2015), we confirmed using RACE-PCR that the *cmaX-cfrX-cmpX* transcription can be initiated from at least one promoter localized 26 nucleotides upstream of the *cmaX* translation initiation codon. Altogether, our results support *cmaX* as a member of AlgU regulon, but also highlight the existence of additional molecular mechanisms that could be involved in the cluster regulation, since a weaker increase of *cfrX* and *cmpX* mRNA levels was also measured under HS. This effect is in line with the previous transcriptomic studies aiming at characterizing *P. aeruginosa* AlgU regulon after a treatment with the D-cycloserine peptidoglycan synthesis inhibitor (Wood and Ohman, 2015), or the response to elevated temperature at 45°C (Chan et al., 2016). If a HS induction of *cmaX* was measured, neither *cfrX* nor *cmpX* were up-regulated, although these three genes have been previously shown to be co-transcribed by RT-PCR (Brinkman et al., 1999). We also showed that under HS the expression profile of the operon is not altered in a *rpoH* genetic background compared to the one obtained in the WT PAO1 strain, suggesting that RpoH is not involved in the HS-inducible expression of *cmaX* in our conditions and comforting the putative AlgU promoter sequence identified upstream the gene. In addition, RpoH does not seem to be involved in the regulation of *cfrX* and *cmpX* as well as *sigX* and *oprF* in our condition in PAO1. However, RNA-seq analyses showed a decrease in the expression of *cfrX-cmpX*, *oprF*, and *sigX*, which could be auto-regulated (Gicquel et al., 2013), as well as an increase of *cmaX* in a *rpoH* overexpressing strain (Schulz et al., 2015), suggesting a potential role of RpoH in the regulation of the operon that would be function of the *P. aeruginosa* strain and/or the condition used. By regulating the expression of many proteases and RNA chaperones such as Hfq that promotes RNA-RNA interactions (Schulz et al., 2015), RpoH could play a key role in the regulation of RNA metabolism by modulating the abundance of many proteins under stress conditions. Since an instability of SigX in a *sigX* overexpressing strain has been reported by Boechat et al. (2013), it could be of interest to better understand RpoH role. Interestingly, the recent development of the GRIL-seq tool to identify the targets of small RNAs has shown that the mRNAs of *cmpX*, but also of *oprF*, *mucA* encoding the AlgU anti-sigma factor, and *amrZ* are all targets of the sRNA PrrF1 *in vivo* (Han et al., 2016). This putative but intriguing regulatory mechanism could be involved in the low but reproducible decrease observed in our study of *sigX* and *amrZ* mRNA levels in *P. aeruginosa* H103 strain when exposed to HS. We propose thus that the interplay between the two major ECFσ controlling the envelope stress response could involve small regulatory RNAs and/or proteases. In line with this hypothesis, the expression of *sigX* was shown to be repressed in *P. putida* KT2440 exposed to elevated pressure, another condition that positively regulates HS but negatively the expression of CS proteins (Follonier et al., 2013).

Another major finding of this study is the increased *sigX* expression following an alteration of the membrane properties or CS. In addition, these results are in agreement with a recent publication, which shows that CspA1 contributes to SigX expression in *Pseudomonas plecoglossicida* (Huang et al., 2020). This result also reinforces the hypothesis of an atypical dependent regulatory mechanism requiring further characterization. Using bioinformatics, one of the main candidates suspected to be involved in SigX activity regulation is CfrX (Staroń et al., 2009). In agreement with previous transcriptomic data (Gicquel et al., 2013; Blanka et al., 2014), we here confirmed that *cfrX* and *cmpX* are two SigX targets. More precisely, we show that their expression is controlled by a large SigX-dependent promoter region, involving a promoter located at −208 nucleotides from the *cfrX* translation start site was identified. Remarkably, this promoter region has a more complex activity pattern than that of *cmaX* in the WT, suggesting the existence of several promoters, possibly under the control of different regulators. Interestingly, a 5′UTR of 13 nucleotides has also been identified by TSS mapping RNA-seq (Schulz et al., 2015), suggesting the existence of at least one additional proximal promoter, whose activity remains, however, to be experimentally validated. No specific TSS for *cmpX* was identified by this technique (Schulz et al., 2015), in agreement with our own unsuccessful attempts, confirming that *cfrX* and *cmpX* are co-transcribed (Brinkman et al., 1999). In view of the mechanisms known to regulate the activity of ECFσ, the role of SigX in the regulation of a large promoter region upstream of the *cfrX-cmpX* transcriptional unit supports the hypothesis of the involvement of CfrX, but also of the mechanosensitive ion channel CmpX in the regulation of SigX activity. In connection with CmpX putative activity as a membrane tension sensor, and the influence of temperature on membrane structure, we artificially altered the membrane fluidity using valinomycin to follow *sigX* response. Exposure to this compound led to an increased membrane stiffness after a relatively short time treatment, validating the chosen experimental strategy. Valinomycin was shown to directly induce alterations in the turnover of phospholipid fatty acids and in the membrane potential in human erythrocytes with respect to its ion channel function (Dise and Goodman, 1985). Following the valinomycin treatment, the *sigX* mRNA relative abundance increased. Similar results were previously observed when *P. aeruginosa* was grown in presence of high sucrose concentration (Bouffartigues et al., 2014), a treatment stabilizing the membrane composition during dehydration (Leslie et al., 1995). Although the molecular mechanism remains to be determined, our data strongly suggest that the membrane stiffness would trigger a bacterial response via SigX. Paradoxically, expression of *sigX* was also increased in response to hypo-osmolarity (Brinkman et al., 1999; Bouffartigues et al., 2012), a condition known for its tendency to increase membrane fluidity (Los and Murata, 2004). However, these effects have not yet been confirmed in *P. aeruginosa* and it would be interesting to evaluate them in hypo-osmotic shock conditions. In view of currently available data on *sigX* expression regulation (Chevalier et al., 2018) and the known effects of CS on bacteria (Barria et al., 2013), other mechanisms could be connected to our observations. Finally, our results confirm the involvement of SigX in controlling membrane fluidity since a *sigX* mutant displays a more rigid membrane, leading to impaired carbon metabolism (Fléchard et al., 2018). As depicted on Figure 6, the highly thermo-regulated processes described in this study highlight an interconnection between the two ECFσ AlgU and SigX to coordinate the CWSR in *P. aeruginosa*, in which the function of the members of the *cmaX-cfrX-cmpX* operon will now have to be clarified with regards to SigX regulation as well as envelope homeostasis. Moreover, we postulate that the expression and/or the activity of these two ECFσ would be coordinated by mechanisms that remain to be identified.

## Materials and Methods

### Bacterial Strains, Media and Growth Conditions

The bacterial strains used in this study are listed in the Supplementary Table S3. Liquid cultures were inoculated in LB medium (171 mM NaCl) at an initial absorbance at 580 nm (A_580_) of 0.08. Bacteria were grown at 37°C with orbital shaking at 180 rpm. Two solid media were used, LB containing 1.5% (w/v) technical agar (AES CHEMUNEX) and *Pseudomonas* Isolation Agar (PIA, Gibco-BRL, Grand Island, NY) for the selection of *Pseudomonas* strains. For CS or HS experiments, cells were grown as described above until the absorbance at 580 nm was between 0.5 and 0.6 and incubated at 4°C into iced water or at 60°C in stove for the indicated time. Control experiments were done by incubating cultures at 37°C in the same conditions. When necessary, the following antibiotics were added at the indicated concentration (μg.mL^–1^): gentamicin (Gm), 50; tetracycline (Tc), 250; carbenicillin (Cb), 300 for *Pseudomonas* strains, and Gm, 15; Tc, 15; ampicillin (Ap), 100 for *E. coli* strains.

### Bacterial Cell Viability Assays by Flow Cytometry

To determine the viability of cells exposed at 37, 4, or 60°C, the LIVE/DEAD^TM^ BacLight^TM^ Bacterial Viability and Counting Kit, for flow cytometry (Invitrogen, Molecular Probes, Carlsbad, CA) was used. Briefly, 10^5^ cells exposed to CS or HS were diluted in 1 mL of PBS and stained following the manufacturer’s instructions. For analysis of the bacterial population using CytoFlex S flow cytometer (Beckman coulter Life science, Indianapolis, United States), red (propidium iodide, PI) and green (SYTO 9) fluorescence was recorded. An aliquot of cells was killed with 100% ethanol to act as a death control. The SYTO9 stained cells were detected by an excitation with 22 mW blue laser at 488 and with emission wavelength at 525 nm (green, with band pass filter of 40 nm), while the PI stained cells were detected by 690 nm (red, with band pass filter of 50 nm).

### Genes Expression Analysis by Real Time Quantitative RT-PCR

Total RNA was extracted from bacterial cultures by the hot acid-phenol method as previously described (Bouffartigues et al., 2012) and tested by PCR for the absence of contaminating DNA using the primers pair FcfrX/RcfrX (Supplementary Table S4). The RNA concentration, the protein and solvent contaminations were determined by measuring the absorbance at 260, 280, and 230 nm, respectively. Total RNA quality was checked on a 2% agarose gel/1X-TAE electrophoresis buffer prior to use for cDNA synthesis. The mRNAs of interest were quantified by real-time PCR amplification of their cDNAs. Each primers pair was validated by verifying that the PCR efficiency was above 0.90, and that a single PCR product with the expected Tm was obtained. PCR were performed on cDNAs at least in triplicate and carried out in SYBR Green PCR Master Mix^TM^ (Applied Biosystems), with 300 nM of each primer (ABI 7500 Fast Q-PCR system, Applied Biosystems). The cDNA-generated signals were internally corrected with the 16S rRNA cDNA signal. The mRNA expression level was calculated by comparing threshold cycle (Ct) of target genes with control sample group and the relative quantification (RQ) data was determined with the 2^–ΔΔ*Ct*^ method using the RQ manager v1.2.1 and the Microsoft-excel based softwares (Muller et al., 2002).

### Bioinformatics Tools

DNA Sequences were extracted from the *Pseudomonas* genome database (Winsor et al., 2016) and analyzed using BPROM bacterial promoter prediction software to predict putative promoters^1^.

### 5′ RACE Assay

Predicted transcription start sites (TSSs) were confirmed using the 5′ RACE (rapid amplification of cDNA ends) procedure (3′/5′ RACE 2nd Generation kit; Roche Molecular Biochemicals) according to the manufacturer’s instructions. Briefly, cDNA was produced from 1 μg of total RNA using gene-specific primer AS1 (Supplementary Table S4) and further purified using the High Pure PCR product purification kit (Roche). Nested PCR was further achieved using a gene-specific AS2 primer (Supplementary Table S4) and oligo(dT) primer. Finally, the PCR product was cloned into pGEM-T Easy vector according to the manufacturer’s instructions (Promega) and 5 clones were sequenced to identify the 5′ end of the specific mRNA (Beckman Coulter Genomics, Villepinte, France).

### Construction of Transcriptional Fusions

Promoter regions of interest were fused to the promoter-less *luxCDABE* cassette in the replicative pAB133 vector (Supplementary Table S3). DNA fragments containing the *cmaX*, *cfrX*, or *cmpX* promoter regions were PCR-amplified with the primers pairs FCmaX-RCmaX, FCFRX- RCFRX, or FCmpX-RCmpX (Supplementary Table S4). The *Sac*I-*Spe*I-digested PCR product was inserted at the same restriction sites into pAB133 yielding pAB*cmaX*, *pABcfr*X, and pAB*cmpX*, respectively. The insert was verified by DNA sequencing.

### Bioluminescence Assay

*P. aeruginosa* strains containing pAB-reporter vectors were grown in covered, white 96-well optiplates with a flat transparent bottom (BD Falcon, San Jose, CA). Bioluminescence and absorbance were simultaneously measured throughout bacterial growth using a thermo-controlled multi-mode plate reader (Xenius, SAFAS, Monaco). The bioluminescence values [in relative light units (RLU) × s^–1^] were divided by the absorbance values at 580 nm of the cultures, yielding relative bioluminescence values (in RLU/sec^∗^A_580_). The relative luminescence values of the negative *P. aeruginosa* control strain harboring the promoter-less vector pAB133 were subtracted from those containing the studied promoter, as described by Bazire et al. (2005). Each set of experiment was performed at least three times.

### Construction of a *P. aeruginosa* H103 *algU* Deletion Mutant

To obtain the Δ*algU* strain isogenic to H103, the gene was replaced by a truncated:Gm disrupted allele carried by the PEXUGL (derivated-PEX100Tlink vector (Quénée et al., 2005, Supplementary Table S3) using the previously described strategy by Bazire et al. (2010). Briefly, this construct was introduced into *E. coli* S17.1 (Simon et al., 1983) and mobilized into *P. aeruginosa* H103 by conjugational mating. Gm-trans conjugates were isolated, the Gm-resistance gene excised and the *algU* deletion confirmed by PCR using the primers pair algU1-algU4 (Bazire et al., 2010).

### Membrane Fluidity Assays by Fluorescence Anisotropy

Bacterial cells grown in LB at 37°C to mid-log phase (A580 = 0.5) were exposed to 45 μM of valinomycin during the indicated time, and harvested by centrifugation, washed twice in sterile Tris-Cl buffer (15 mM, pH 7), and suspended in the same buffer to OD_580_ = 0.2. Membrane fluidity was measured using the probe 1,6-diphenyl-1,3,5-hexatriene (DPH, Sigma Aldrich) or its derivative N,N,N-Trimethyl-4-(6-phenyl-1,3,5-hexatrien-1-yl)phenylammonium p-toluenesulfonate (TMA-DPH) as previously described (Baysse et al., 2005).

### Proteomic Analysis

Impact of a CS on the proteome of *P. aeruginosa* H103 was performed by 2D-electrophoresis as previously described by Massier et al. (2012) with minor modifications. Briefly, total proteins were extracted from cells, exposed or not to a CS during 30 min, as follow: bacteria were harvested from 10 mL of three independent control or CS-treated cultures by centrifugation (7,500 g for 20 min at 4°C) and suspended in 5 mL of lysis buffer (30 mM Tris-Cl pH8 and 0.1 mM phenylmethanesulfonylfluoride) prior to sonication. 350 μg of proteins were solubilized in 325 μL of re-hydratation buffer (7 M urea, 2 M thiourea, 4% CHAPS 3-((3-cholamidopropyl) dimethylammonio)-1-propanesulfonate, Bio-Rad Laboratories], 65 mM DTT, 0.5% carrier ampholytes mixture pH3-10 (Bio-Rad Laboratories) prior to loading on ReadyStrip^TM^ IPG strip (17 cm, pH 4–7, Bio-Rad Laboratories). After a passive rehydration step during 16 h, isoelectric focusing parameters were set as 50 μA/strip at 20°C and carried out using the following conditions: 0–100 V linear ramp for 45 min, 100–250 V rapid ramp for 15 min, 250–10,000 V gradual ramp for 2 h 45 and hold at 10,000 V until a total of 52,000 V × h was reached. The proteins were separated by SDS-PAGE 10% and coomassie blue stained. Two gels per protein sample were performed and scanned using a GS-800 densitometer (Bio-Rad Laboratories) to allow normalization and quantification of reproducible spots detected with PDQuest 2-DE analysis 7.4.0 software (Bio-Rad Laboratories). Protein spots were excised from gels and characterized through matrix-assisted laser desorption ionization time of flight mass spectrometry (MALDI-TOF/MS) analysis. Search parameters and statistical analyses of the sequences for proteins identification were determined by the probability-based Mowse score offered by MASCOT software as described previously (Barbey et al., 2012) using SwissProt 2020. The mass spectrometry proteomics data have been deposited to the ProteomeXchange Consortium via the PRIDE (Perez-Riverol et al., 2019) partner repository with the dataset identifier PXD021299.

### Statistics and Reproducibility

All experiments were carried out at least three times independently. For RT-qPCR experiment *P*-values were calculated with the two-tailed *t*-test using qstats a complementary function of the Microsoft-excel based softwares previously mentioned (Muller et al., 2002; Figures 1, 3B,C, 4A,C, 5B,C and Supplementary Figure S2). Live/dead experiments, 2D-E and reporter-based assays were repeated three times with similar results as specify by the indicated standard deviation (Figures 2D, 3A, 5A and Supplementary Figures S1–S3). Box plot and statistical analyses for fluorescence anisotropy data were performed with Qtiplot software (Figure 4B and Supplementary Figure S4).

## Data Availability Statement

The mass spectrometry proteomics data have been deposited to the ProteomeXchange Consortium via the PRIDE (63) partner repository with the dataset identifier PXD021299.

## Author Contributions

EB and SC designed the research and wrote the manuscript with input from all authors. EB, IS, OM, DT, JO, AD, AT, RD, CA, and MN performed the experiments.

## Conflict of Interest

The authors declare that the research was conducted in the absence of any commercial or financial relationships that could be construed as a potential conflict of interest.

## Footnotes

1 http://www.softberry.com/berry.phtml?topic=bprom&group=programs&subgroup=gfindb

## Supplementary Material

Supplementary Figure 1 Flow cytometry analysis of *P. aeruginosa* H103 viability exposed or not to CS or HS. Bacteria were grown in LB until the mid-log growth phase and subjected to CS or HS as indicated. 10^5^ cells were stained using Syto9 and PI and then analyzed by flow cytometry. **(A)** Cytograms are representative of three independent experiments. Live and dead cells emitting a green or a red fluorescence are indicated within a red or purple frame, respectively. The green frame corresponds to cells with membrane damages. The mean percentage of live, dead and injured cells is indicated in each frame. **(B)** Histograms representing percent of cell viability from the experiments of live/dead assays (*n* = 3).

Supplementary Figure 2 Effect of HS and CS on *algU* expression and activity. Black and gray bars show means normalized expression of *algU* and its targets after HS or CS relatively to the control condition at 37°C as calibrator (white and dotted bars). NT: not treated. Error bars indicate SEM, *n* = 4. Significant differences between the sets of normalized expression obtained in each stress condition were calculated against the those obtained at 37°C using a two-tailed *t*-test, and *P-*value are indicated by ^∗^, ^∗∗^, or ^∗∗∗^ if < 0.05, 0.001, or 0.0001, respectively. The symbol ° is indicated for non-significant results.

Supplementary Figure 3 RpoH is not involved in the *cmaX, cfrX, and cmpX* regulation in response to HS in PAO1. Means of normalized expression for *algU* targets (red frame), the predicted operon (*cmaX-cfrX-cmpX*) and *sigX* in PAO1 (red line) or in an isogenic *rpoH* mutant exposed (dashed back bars) or not (dashed white bars) to HS.

Supplementary Figure 4 2-DE total protein profiles of *P. aeruginosa* exposed to CS from 37 to 4°C or not (control). Gels are Coomassie blue stained and are representative of three independent proteins extractions. The protein spots satisfying both statistical biological variation analysis and mass spectroscopy identification scores were numbered according to Table 1 and Supplementary Table 2.

Supplementary Figure 5 c-di-GMP level quantification by LC-MS/MS in H103 strain exposed or not to a CS. Intracellular c-di-GMP was extracted in H103 strain exposed (square) or not (point) to CS and quantified LC-MS/MS. Briefly, the absorbance at 580 nm of cultures were recorded and cells from 2 mL of culture were collected. Then cells were harvested by centrifugation, washed in 1 mL of sterile physiological water and c-di-GMP was extracted twice with extraction buffer without internal standard and quantified as previously described (Bouffartigues et al., 2015; Strehmel et al., 2015).

Supplementary Figure 6 Effect of a sublethal dose of valinomycin on membrane fluidity measured by fluorescence polarization in stationary growth phase. TMA-DPH and DPH anisotropies were used to evaluate the effect of 45 μM valinomycin on membrane fluidity 22 h after the addition of the membrane acting-agent to mid-log phase cultures. Values obtained from unexposed cultures are indicated as NT: not treated. Box plots correspond to each sets of data from three independent experiments. Means, medians and min/max anisotropies values are indicated by squares, lines and crosses, respectively. Significant differences between each condition were calculated using a two-tailed *t*-test, and *P*-value are indicated by ^∗^ if < 0.05.

Supplementary Table 1 Normalized expression of the studied genes under HS or CS in the indicated genetic backgrounds relative to *P. aeruginosa* H103 at 37°C. Values are means ± SEM for at least three independent experiments.

Supplementary Table 2 Characteristics of proteins identified by Maldi-TOF analysis. MS and MS/MS correspond to the mass spectrometry method used for the protein identification.

Supplementary Table 3 Bacterial strains and plasmids used in this study. Cb^r^, carbenicillin resistance; Gm^r^, gentamycin resistance; Tc^r^, tetracycline resistance.

Supplementary Table 4 Primer sequences of the indicated genes used for quantitative RT-qPCR reactions, transcription start sites identification and transcriptional fusions construction.
