# Supplementary material for: The Temperature-Regulation of Pseudomonas aeruginosa cmaX-cfrX-cmpX Operon Reveals an Intriguing Molecular Network Involving the Sigma Factors AlgU and SigX
Source: Front Microbiol. 2020 Oct 21;11:579495. doi: 10.3389/fmicb.2020.579495 (PMC7641640; doi:10.3389/fmicb.2020.579495)
Supplement: Supplementary Figure 1 — Flow cytometry analysis of P. aeruginosa H103 viability exposed or not to CS or HS. Bacteria were grown in LB until the mid-log growth phase and subjected to CS or HS as indicated. 105 cells were stained using Syto9 and PI and then analyzed by flow cytometry. (A) Cytograms are representative of three independent experiments. Live and dead cells emitting a green or a red fluorescence are indicated within a red or purple frame, respectively. The green frame corresponds to cells with membrane damages. The mean percentage of live, dead and injured cells is indicated in each frame. (B) Histograms representing percent of cell viability from the experiments of live/dead assays (n = 3). [file Data_Sheet_1.pdf]

The temperature-regulation of *Pseudomonas aeruginosa cmaX-cfrX-cmpX* operon reveals an intriguing molecular network involving the sigma factors AlgU and SigX

Emeline Bouffartigues, Ishac Si Hadj Mohand, Olivier Maillot, Damien Tortuel, Jordane Omnes, Audrey David, Ali Tahrioui, Rachel Duchesne, Cecil Onyedikachi Azuama, Michael Nusser, Gerald Brenner-Weiss, Alexis Bazire, Nathalie Connil, Nicole Orange, Marc GJ Feuilloley, Olivier Lesouhaitier, Alain Dufour, Pierre Cornelis, Sylvie Chevalier

SUPPLEMENTARY DATA

Table of contents

	Pages
**Table S1:** Normalized expression of the studied genes under HS or CS in the indicated genetic background relative to *P. aeruginosa* H103 at 37°C	**2**
**Table S2:** Characteristics of proteins identified by Maldi-TOF analysis	**3**
**Table S3:** Bacterial strains and plasmids used in this study	**4-5**
**Table S4:** Primer sequences of the indicated genes used for quantitative RT-qPCR reactions, transcription start sites identification and transcriptional fusions construction	**6-7**
**Fig. S1:** Flow cytometry analysis of *P. aeruginosa* H103 viability exposed or not to CS or HS	**8**
**Fig. S2:** Effect of HS and CS on *algU* expression and activity	**9**
**Fig. S3:** RpoH is not involved in the *cmaX, cfrX and cmpX* regulation in response to HS in mPAO1.	**10**
**Figure S4:** 2-DE total protein profiles of *P. aeruginosa* exposed to CS from 37°C to 4°C or not (control)	11
**Fig. S5:** c-di-GMP level quantification by LC/MS/MS in H103 strain exposed (square) or not to a CS (point)	**12**
**Fig. S6:** Effect of a sublethal dose of valinomycin on membrane fluidity measured by fluorescence polarization in stationary growth phase	**13**

Table S1: Normalized expression of the studied genes under HS or CS in the indicated genetic backgrounds relative to *P. aeruginosa* H103 at 37°C.

		Genetic background		
**Target genes**	**Treatment**	**H103**	**Δ*algU***	**Δ*sigX***
cmaX	HS	5.9 ± 1	0.9 ± 0.2	
	CS	1 ± 0.4		
cfrX	HS	1.7 ± 0.3	1.3 ± 0.4	
	CS	3.6 ± 0.08		1.3 ± 0.1
cmpX	HS	2.1 ± 0.4	1.3 ± 0.4	
	CS	3.1 ± 0.3		1. ± 0.3
algU	HS	2.9 ± 1.7		
	CS	1.4 ± 0.7		
algD	HS	19.4 ± 6.3	6 ± 0.5	
	CS	1.5 ± 0.3		
rpoH	HS	4 ± 1.2	1.4 ± 0.3	
	CS	1.3 ± 0.4		
oprF	HS	1.6 ± 0.1	0.8 ± 0.2	
	CS	1 ± 0.1		
amrZ	HS	0.4 ± 0.1	0.3 ± 0.03	
	CS	1± 0.3		
sigX	HS	0.8 ± 0.1	0.6 ± 0.1	
	CS	2 ± 0.6		0.9 ± 0.2
accA	HS	1 ± 0.1		
	CS	1.8 ± 0.2		1.4 ± 0.1
accB	HS			
	CS	2.7 ± 0.3		1.9 ± 0.8
**PA5174**	HS			
	CS	2.5 ± 0.8		1.2 ± 0.5
psrA	HS			
	CS	0.5 ± 0.1		0.2 ± 0.02

Table S2: Characteristics of proteins identified by Maldi-TOF analysis.

Spot number	RefSeq	PA number	Protein name	Theorical MW(kDa)/pI	method	Protein Score	Number of Matched peptides	Sequence coverage (%)
1	NP_251641.1	PA2951	EtfA	31.4/4.75	MS/MS	54	1	5
2	NP_259243.1	PA0552	Pgk	40.4/5.06	MS/MS	123	2	5
3	NP_251641.1	PA2953	EtfD	59.9/5.6	MS/MS	42	1	1
4	NP_253858.1	PA5171	ArcA	46.4/5.5	MS	167	12	32
5	NP_249556.1	PA0865	Hpd	39.9/4.89	MS	133	12	29
6	NP_252329.1	PA3639	AccA	34.9/5.15	MS	84	7	26
7	NP_252345	PA3655	Tsf	30.7/4.95	MS	135	9	25
8	NP_252955	PA4265	TufA	43.3/5.03	MS	133	9	29
9	NP_253428.1	PA4740	Pnp	75.4/4.81	MS	112	9	17

Table S3: Bacterial strains and plasmids used in this study.

Cb^r, carbenicillin resistance ; Gm^r, gentamycin resistance ; Tc^r, tetracycline resistance.

Strains/plasmids	Relevant characteristic(s)	Sources
**Strains**		
E. coli		
JM109	Cloning host	Promega
S17.1	Conjugation strain	Simon et al., 1983
Pseudomonas aeruginosa		
H103	PAO1 derivative	Hancock, R. E. & Carey, 1979
mPAO1	PAO1 derivative	Jacobs, M. A. et al., 2003
ΔsigX (PAOSX)	PAO1 H103 *ΔsigX*	Bouffartigues et al., 2012
ΔrpoH	Transposon mutant *rpoH*::IS;*phoA*/hah-Tc^r	Jacobs, M. A. et al., 2003
ΔalgU	PAO1 H103 *ΔalgU*	This study
**Plasmids**		
pAB133	Vector for transcriptional fusion analyzes. Gm^r	Bazire et al., 2005
pAB*cmaX*L	pAB-P*cmaX-luxCDABE*. Gm^r	This study
pAB*cfrX*L	pAB-P*cfrX-luxCDABE*. Gm^r	This study
pAB*cmpX*L	pAB-P*cmpX-luxCDABE*. Gm^r	This study
pEXUGL	pEXalgU with *lox-aacC1-lox* cassette. Ap^r, Gm^r	Bazire et al., 2010
pCM157	*cre* expression vector, Tc^r	Quenée et al., 2005

References for Table S3

Bouffartigues, E. *et al.* Transcription of the oprF gene of Pseudomonas aeruginosa is dependent mainly on the SigX sigma factor and is sucrose induced. *J. Bacteriol.* **194**, 4301–4311 (2012).

Hancock, R. E. & Carey, A. M. Outer membrane of Pseudomonas aeruginosa: heat- 2-mercaptoethanol-modifiable proteins. *J. Bacteriol.* **140**, 902–910 (1979).

Jacobs, M. A. *et al.* Comprehensive transposon mutant library of Pseudomonas aeruginosa. *Proc. Natl. Acad. Sci. U. S. A.* **100**, 14339–14344 (2003).

Bazire, A. *et al.* Osmotic stress and phosphate limitation alter production of cell-to-cell signal molecules and rhamnolipid biosurfactant by Pseudomonas aeruginosa. *FEMS Microbiol. Lett.* **253**, 125–131 (2005).

Quénée, L., Lamotte, D. & Polack, B. Combined sacB-based negative selection and cre-lox antibiotic marker recycling for efficient gene deletion in pseudomonas aeruginosa. *BioTechniques* **38**, 63–67 (2005).

Bazire, A. *et al.* The sigma factor AlgU plays a key role in formation of robust biofilms by nonmucoid Pseudomonas aeruginosa. *J. Bacteriol.* **192**, 3001–3010 (2010).

Simon, R., Priefer, U. & Pühler, A. A Broad Host Range Mobilization System for In Vivo Genetic Engineering: Transposon Mutagenesis in Gram Negative Bacteria. *Nat. Biotechnol.* **1**, 784–791 (1983).

Table S4: Primer sequences of the indicated genes used for quantitative RT-qPCR reactions, transcription start sites identification and transcriptional fusions construction.

PA number	Gene name	Primer name	Sequence (5'- 3')
**RT-qPCR**			
PA1773	*cmaX*	PA1773	AAAGGGCCGAAGACAGCAT
		PA1773	CGCGATCGGTCAGGTAGTG
PA1774	*cfrX*	PA1774	CTGCGGGACCTCGTCAAG
		PA1774	GCCGACCTGGCGATTG
PA1775	*cmpX*	FPA1775	GGCAGATCATTGCAGGAATCTAC
		RPA1775	TCTCTTCAATAGTGCCTTCAACGT
PA1609	*fabB*	F1609	CCTCGGCAATGACAAAGACA
		R1609	GTAGGACGGATTGAAGCGAATG
PA0762	*algU*	FalgU	TACCTGGCTGTATCGGATCG
		RalgU	GAAGAACTCCGCATCCTCTG
PA3540	*algD*	FPA3540	GGGCTATGTCGGTGCAGTATG
		RPA3540	GCGACTTGCCCTGGTTGAT
PA	*rpoH*	FrpoH	CGCAAACCTGGAAGCCTA
		RrpoH	GAACAACGAAGCGCAGGT
PA1777	*oprF*	FPA1777	GCGTACAGCTGGACATGAAG
		RPA1777	TTCATGAAGTCAGCCAGGTTCTT
PA3385	*amrZ*	FamrZ	CGTGAGCAGATCGCAGAA
		RamrZ	GCTCGTGCAGGCTGAGTT
PA1776	*sigX*	FPA1776	AATTGATGCGGCGTTACCA
		RPA1776	CCAGGTAGCGGGCACAGA
PA3639	*accA*	FaccA	TCTTCGGCAATCTGACCAGTT
		RaccA	GTAGCCGATGTAGTCGAGGGTA
PA4847	*accB*	FaccB	AAGCCATGAAGATGATGAACC
		RaccB	CGTTCTCCACCAGGATCGA
PA5174	*fabY*	FPA5174	AGGGCGACCTGGAGATCAT
		RPA5174	GCGCGTCCTTCTTGTATACCA
PA3006	*psrA*	FpsrA	CGATTTCGGCGTGAACACTT
		RpsrA	CGAAGAACGGCACCATCAG
PA0668.1	*16SrRNA*	F16SRNA	AACCTGGGAACTGCATCCAA
		R16SRNA	CTTCGCCACTGGTGTTCCTT
**RACE-PCR**			
PA1775	*cmpX*	ASP1cmpX	CCTTCAACGTCACCCACTTT
		ASP2cmpX	AACACCTTGGGCAGGTACAG

PA1774	*cfrX*	AS1cfrX	GCAGAAGATGCGTTTTTCG
		ASP2cfrX	GCGGAAATACGGGAAACC
PA1773	*cmaX*	ASP1cmaX	CAGGTAGTGCGCGAGGTAGT
		ASP2cmaX	CGGATTCAGGTTCACTCCAC
**Transcriptional fusions**			
**PA1773	***cmax*	FCmaX	*taataagagctcGTTCGTGTATGCCGACAACA
		RCmaX	*taataactagttGCGCTTCCCTTGGTAAAAC
**PA1774	***cfrX*	FCFRX	*taataagagctcGAGTTGATACGGGAGCGCAT
		RCFRX	*taataactagttCATGTCTGTACGAGGGAGTG
**PA1775	***cmpX*	FCmpX	*taataagagctcCTGCGGGACCTGCTCAAG
		RCmpX	*taataactagttCACTCAGCCTTGTCAGC

* Nucleotides not in the chromosomal sequence are indicated in lower case.

** Gene localized downstream the PCR-amplified DNA fragment

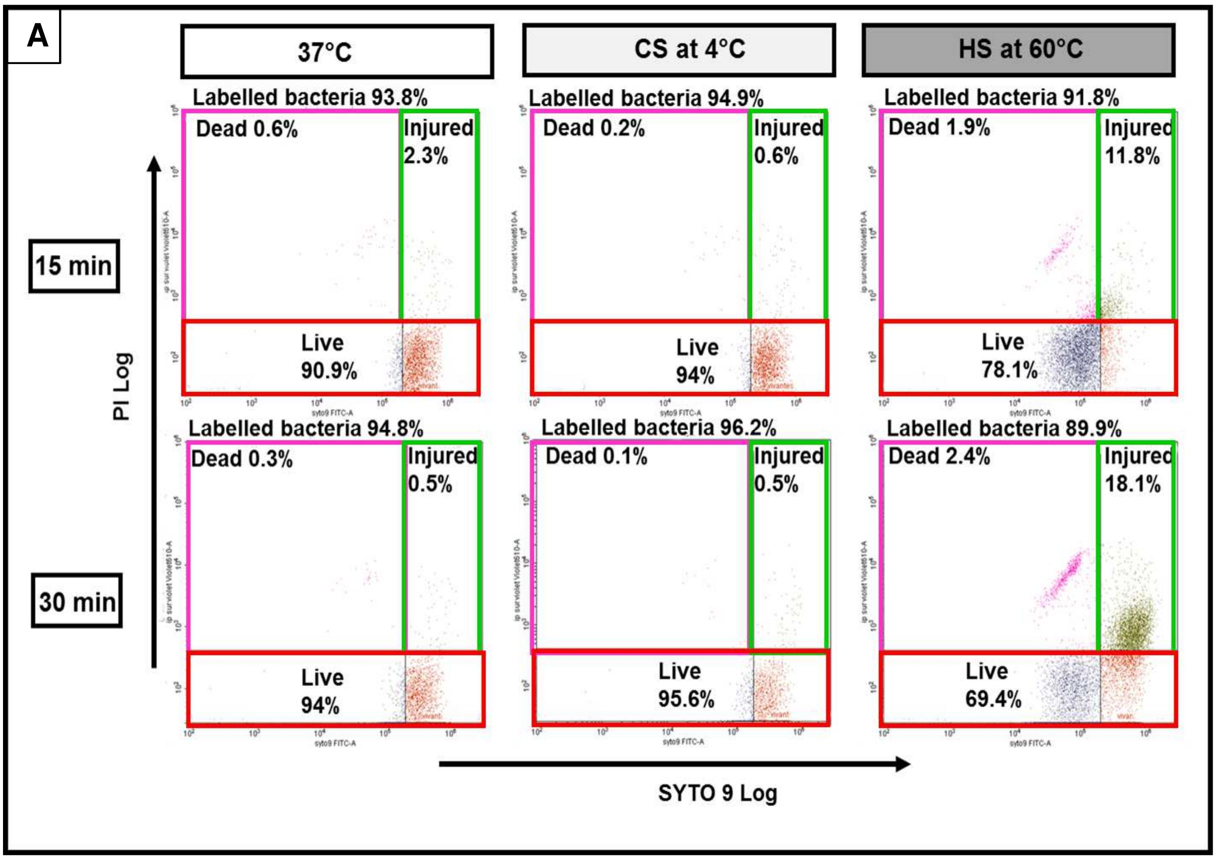

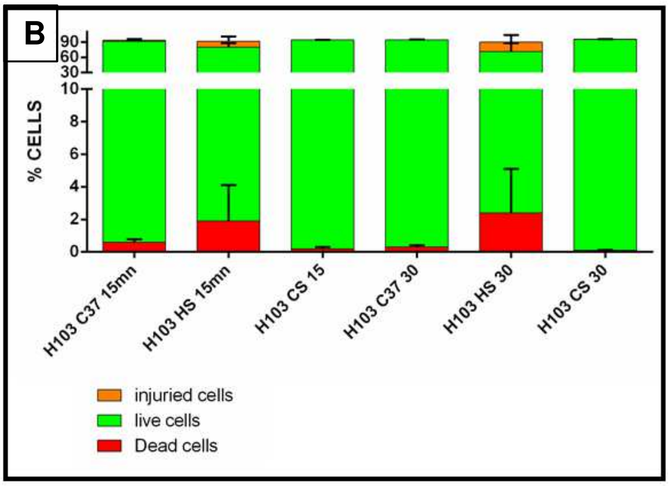

Fig. S1: Flow cytometry analysis of *P. aeruginosa* H103 viability exposed or not to CS or HS.

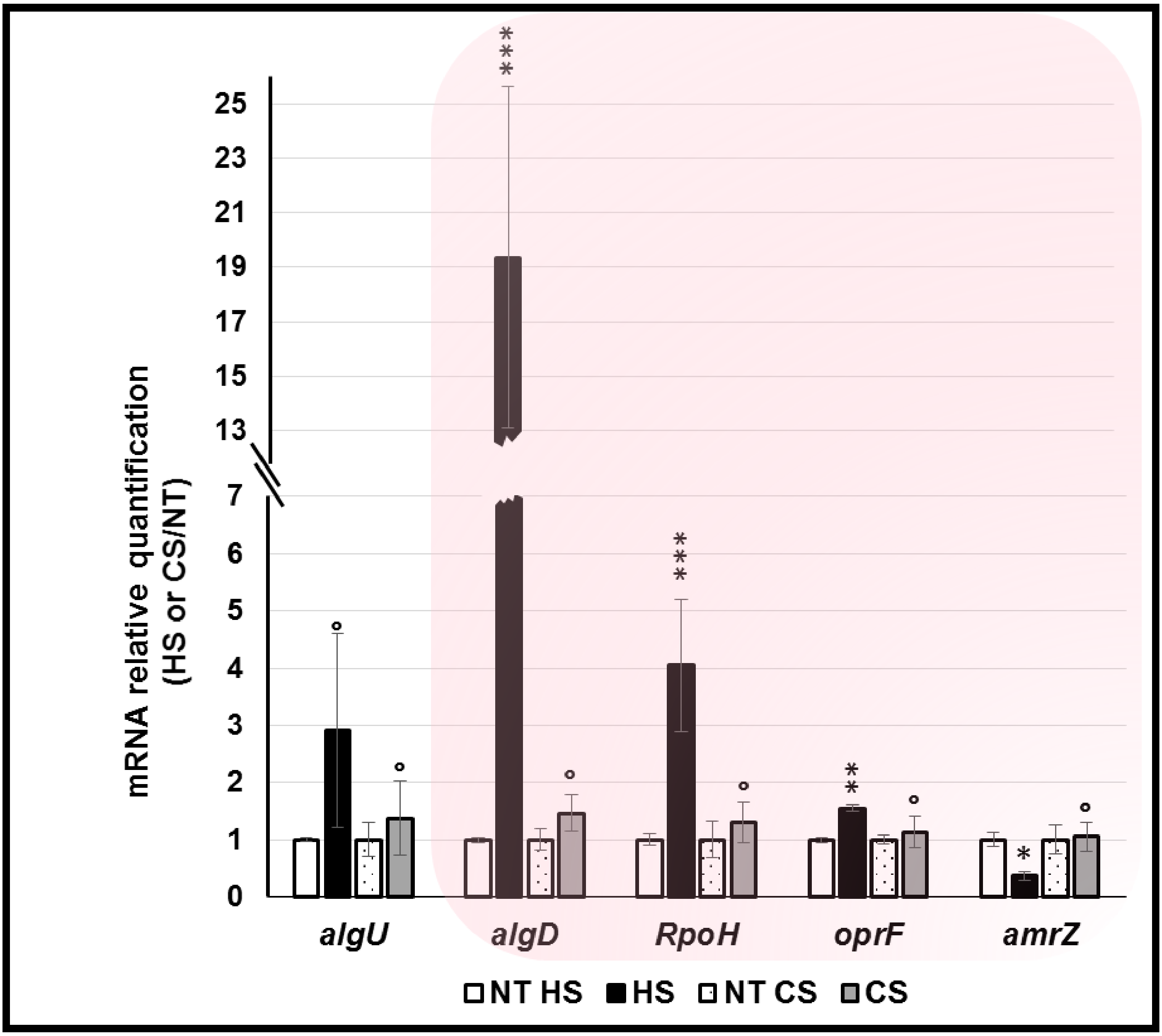

Fig. S2: Effect of HS and CS on *algU* expression and activity.

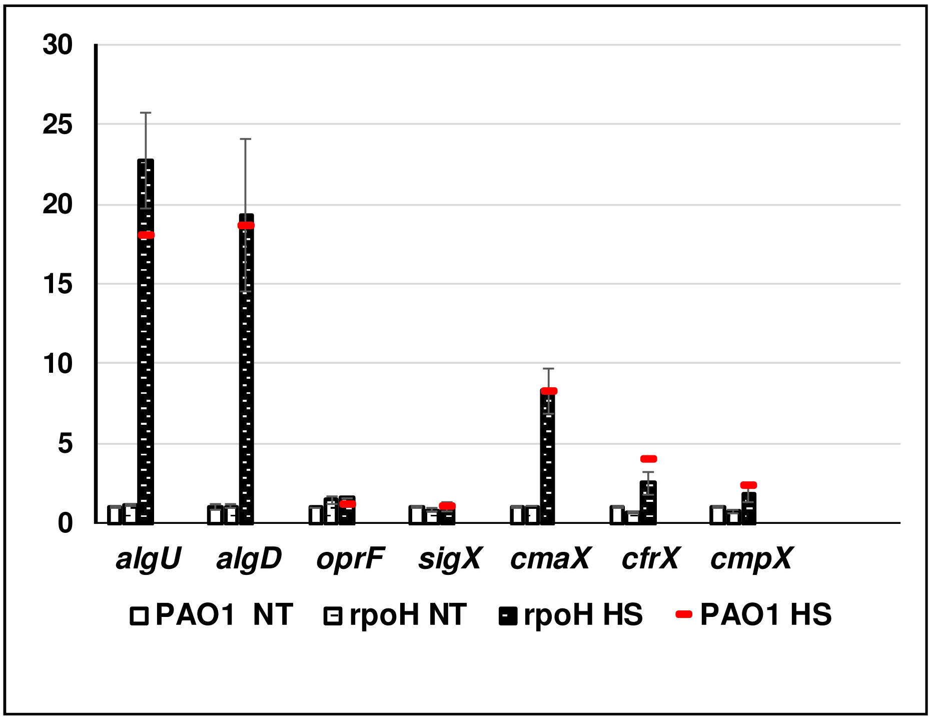

Fig. S3: RpoH is not involved in the *cmaX, cfrX and cmpX* regulation in response to HS in PAO1.

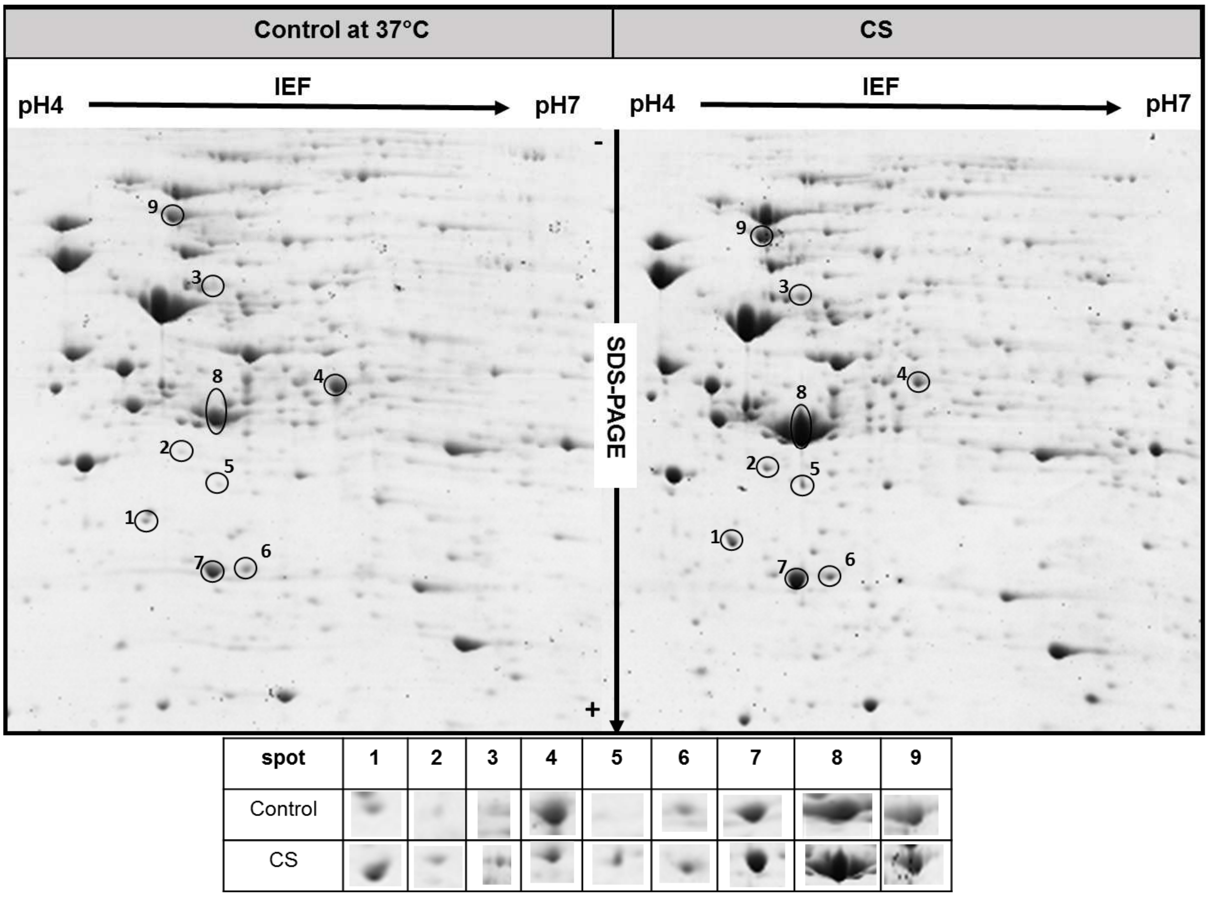

Fig. S4: 2-DE total protein profiles of *P. aeruginosa* exposed to CS from 37°C to 4°C or not (control).

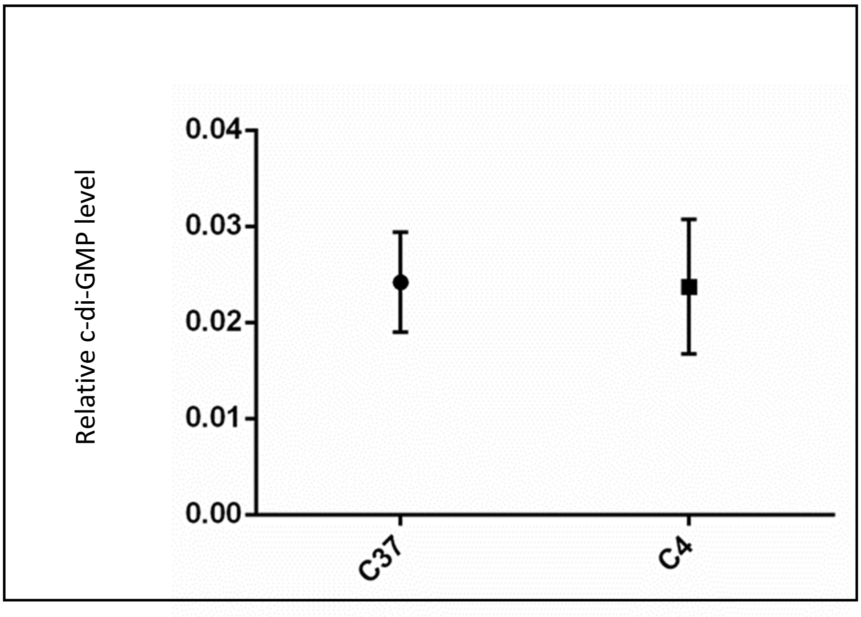

Fig. S5: c-di-GMP level quantification by LC-MS/MS in H103 strain exposed (square) or not to a CS (point).

References for Supplementary Fig. S5

Strehmel, J. *et al.* Sensor kinase PA4398 modulates swarming motility and biofilm formation in Pseudomonas aeruginosa PA14. *Appl Environ Microbiol.* 81(4):1274-85. (2015).

Bouffartigues, E. *et al.* The absence of the *Pseudomonas aeruginosa* OprF protein leads to increased biofilm formation through variation in c-di-GMP level. *Front Microbiol.*; 6: 630. (2015).

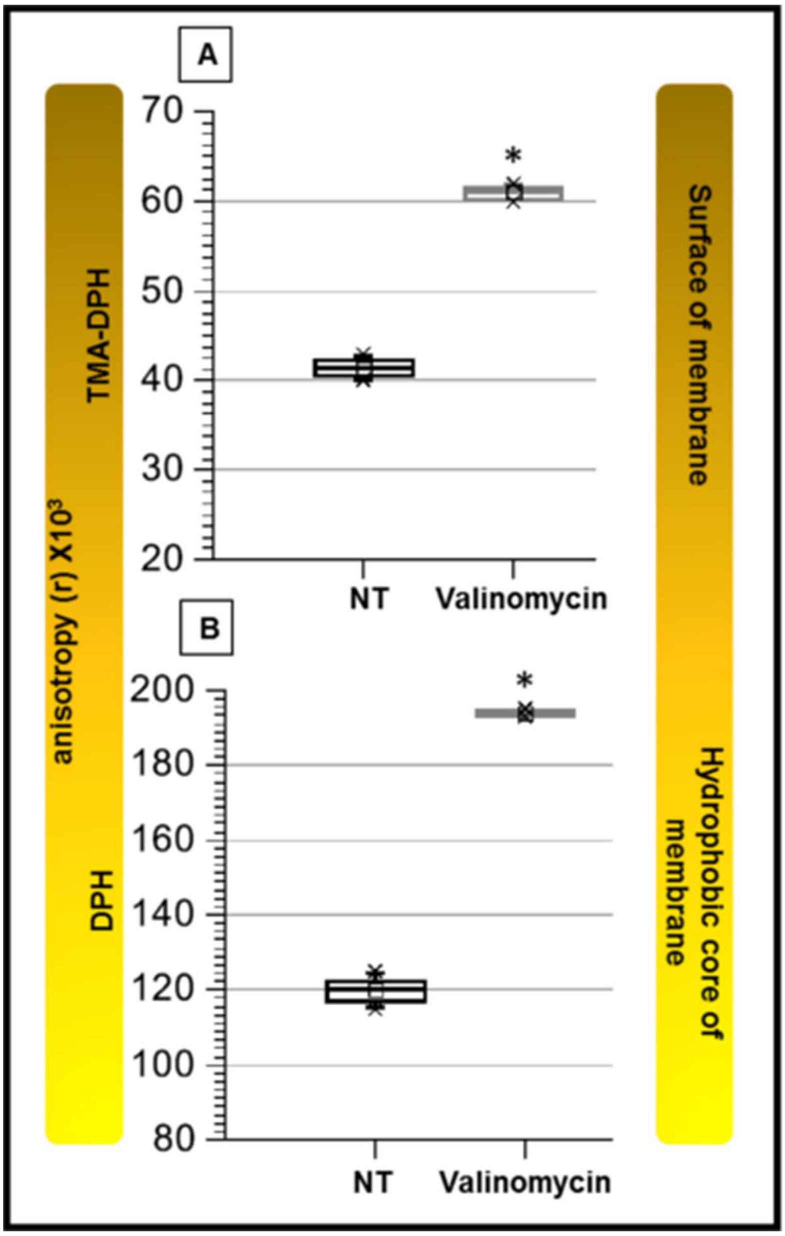

Fig. S6: Effect of a sublethal dose of valinomycin on membrane fluidity measured by fluorescence polarization in stationary growth phase.
